# Propelling a Course-Based Undergraduate Research Experience Using an Open-Access Online Undergraduate Research Journal

**DOI:** 10.3389/fmicb.2020.589025

**Published:** 2020-11-23

**Authors:** Evelyn Sun, Marcia L. Graves, David C. Oliver

**Affiliations:** Department of Microbiology and Immunology, University of British Columbia, Vancouver, BC, Canada

**Keywords:** course-based undergraduate research experience, undergraduate research journal, scientific enculturation, pedagogy, curriculum, molecular microbiology, STEM-science technology engineering mathematics

## Abstract

The University of British Columbia has developed a course-based undergraduate research experience (CURE) that engages students in authentic molecular microbiology research. This capstone course is uniquely built around an open-access online undergraduate research journal entitled Undergraduate Journal of Experimental Microbiology and Immunology (UJEMI). Students work in teams to derive an original research question, formulate a testable hypothesis, draft a research proposal, carry out experiments in the laboratory, and publish their results in UJEMI. The CURE operates in a feed forward manner whereby student-authored UJEMI publications drive research questions in subsequent terms of the course. Progress toward submission of an original manuscript is scaffolded using a series of communication assignments which facilitate formative development. We present a periodic model of our CURE that guides students through a research cycle. We review two ongoing course-based projects to highlight how UJEMI publications prime new research questions in the course. A journal-driven CURE represents a broadly applicable pedagogical tool that immerses students in the process of doing science.

## Introduction

Becoming a scientist is a complex endeavor that requires multiple levels of development. An essential goal for any successful undergraduate program in STEM is to provide opportunities for students to develop skills in the context of being able to do real-world science (Laursen et al., 2010; American Association for the Advancement of Science, 2011; Feldman et al., 2013). To support students as scientists in training, activities in the curriculum ought to ensure that students acquire technical skills, the ability to read and interpret scientific literature, learn how to design experiments, document observations, analyze and interpret data, and have the opportunity to disseminate research findings (Coil et al., 2010). These fundamental skills form a foundation to support higher order activities including innovation, teamwork, self-authorship, expert thinking, collaboration, and meaningful engagement with the scientific community. Collectively, this developmental process can be described as scientific enculturation (Florence and Yore, 2004; Auchincloss et al., 2014; Linn et al., 2015).

Scientific enculturation requires opportunities for students to “do science” (Linn et al., 2015). Research experiences and mentorship from scientists are needed for students to acquire on the ground training, disciplinary knowledge and understanding of the scientific method (Linn et al., 2015; Estrada et al., 2018). Several types of undergraduate research opportunities have been documented to provide a range of different scientific experiences (Lopatto, 2004, 2010; Seymour et al., 2004; Russell et al., 2007; Sadler et al., 2010; Linn et al., 2015; Robnett et al., 2015). Credit-based undergraduate research opportunities include protocol-driven teaching laboratories, inquiry-based teaching laboratories such as course-based undergraduate research experience (CUREs), and research internships. While protocol-driven teaching labs generally involve activities where the experimental results are known at the outset, at least to the instructor (Weaver et al., 2008), CUREs and research internships tend to address novel research questions where the experimental outcome is usually unknown (Auchincloss et al., 2014; Beck et al., 2014). Research internships, often called directed studies or honors thesis projects, typically have a one-to-one structure where an undergraduate student mentee is paired with a more senior scientist as a mentor (Shapiro et al., 2015). With capable mentors, internships can provide high quality research experiences; however, because mentor:mentee pairings tend to be self-selecting, student diversity, and equitable access can be limited (Bangera and Brownell, 2014). In contrast, CUREs are designed to be scalable and accessible by accommodating a few to several hundred student mentees to one or more faculty mentors (Linn et al., 2015). The course-based nature of CUREs also means that lectures and tutorials can be paired with research activities to provide consistent training in fundamental research skills. CUREs are a rapidly growing pedagogical model for teaching science curricula to promote enculturation and scientific identity among all students in a program, and not an exclusive few (Bangera and Brownell, 2014; Esparza et al., 2020).

Auchincloss et al. (2014) proposed that science-based CUREs can be defined by five main domains in which students: (1) engage in scientific practices, which include technical skills development and the use of the scientific method, (2) experience discovery, as the outcome of an experiment is not known by the students or the instructor at the outset, (3) pursue research questions with broad relevance and meaning beyond the classroom setting, (4) collaborate with their peers, as fellow scientists, and sometimes with practicing scientists in the broader community, and (5) iterate, as experiments are repeated, refined, and cross-examined to generate more robust models and concrete knowledge. Taken together, these domains provide students with an experience that integrates the complex facets of doing authentic research (Brownell et al., 2015). As a result, positive outcomes of CUREs on student development have been documented in several areas of research competency including science identity and confidence, content knowledge, and science literacy (Brownell et al., 2015; Olimpo et al., 2016).

A broad range of science-based CUREs have been developed within disciplines (e.g., biology, chemistry, physics, mathematics, geography) as well as across disciplines (Brownell et al., 2015; Kerr and Yan, 2016; Sarmah et al., 2016; Shanle et al., 2016; Alford et al., 2017; Ballen et al., 2017; Ayella and Beck, 2018; Light et al., 2019; Shelby, 2019; Stoeckman et al., 2019; Wolkow et al., 2019). Bhattacharyya et al. (2020) showcase the wide range of diversity in CURE design (Bhattacharyya et al., 2020). Some CURE courses focus on one main biological model such as expression of p53 tumor suppressor gene in yeast (Brownell et al., 2015), protein interactions with Mer tyrosine kinase (Shelby, 2019), mutagenesis of lactate dehydrogenase (Ayella and Beck, 2018), or the effect of nicotine and caffeine on the development of zebrafish (Sarmah et al., 2016). Some involve students collaborating with an outside institution to conduct their research projects such as the Rosetta Research Experience for Undergraduates where students undertake their CUREs outside of the institution following a 2 weeks programming boot camp (Alford et al., 2017). Others involve consecutive CURE courses taken throughout a student’s degree that progressively building a single research topic (e.g., antibiotic resistance) (Light et al., 2019).

Here we review a capstone CURE developed at the University of British Columbia that centers around student-driven microbiology-based projects, and culminates in the generation of original research articles published in an online undergraduate research journal titled the Undergraduate Journal of Experimental Microbiology and Immunology (UJEMI). We describe the structure and function of our CURE model and discuss UJEMI as a tool with the potential to objectively assess student development and observe the process of scientific enculturation. We hope that insights gleaned from our experiences may be helpful to others seeking to design and understand the pedagogical value of CUREs.

## An Undergraduate Research Journal-Driven Cure

The University of British Columbia’s (UBC) Point Grey campus located in Vancouver, Canada is a large research intensive post-secondary institution which serves over 45,000 undergraduate students and 10,000 graduate students annually (The University of British Columbia, 2020). Since 2001, UBC has been developing a capstone molecular microbiology CURE that serves students in the final year of their 4 year undergraduate program offered by the Department of Microbiology and Immunology. Initially starting out as an optional course enrolling a few students, the course is now required for graduation and has grown to accommodate up to 60 students per semester, totaling approximately 120 students per academic year. Prior to enrolling in the CURE, students are required to complete two lab courses. One is a traditional, protocol-driven lab while the other is a guided inquiry-based lab; together they provide students with fundamental knowledge and skills required to begin working independently in a molecular microbiology laboratory. Resourced with a single instructor and one to two graduate student teaching assistants, the CURE unfolds over 16 weeks (September–December or January–April).

The course is equipped with four learning centers: an interactive classroom lecture (1.5–3 h per week), a team-based meeting (1 h per week), web-based resources including classroom management tools for communication and an open-access undergraduate research journal^1^, and a wet-bench research laboratory outfitted with the majority of the tools necessary to conduct microbiology and molecular biology which is open to students throughout the week. In addition, students are encouraged to interact with researchers working in grant-funded laboratories at UBC, which increases the scope and breadth of the scientific and technological resources available in the course.

The primary instructor of the CURE manages our undergraduate research journal, UJEMI. The structure and function of UJEMI have been previously described (Sun et al., 2020). Briefly, the CURE instructor mentors graduate student editors employed as teaching assistants over the summer months to administer a student-centered peer-review experience, and prepare the manuscripts for online publication (Sun et al., 2020). In addition to papers generated from research conducted in our CURE, UJEMI invites submissions from undergraduate students doing scientific research projects in microbiology and/or immunology at accredited universities around the world. Taken together, UJEMI provides a platform for undergraduate researchers to participate in the authentic process of research dissemination as published authors, and the novel findings published as UJEMI articles drive new research questions in our CURE term after term.

**FIGURE 1:**
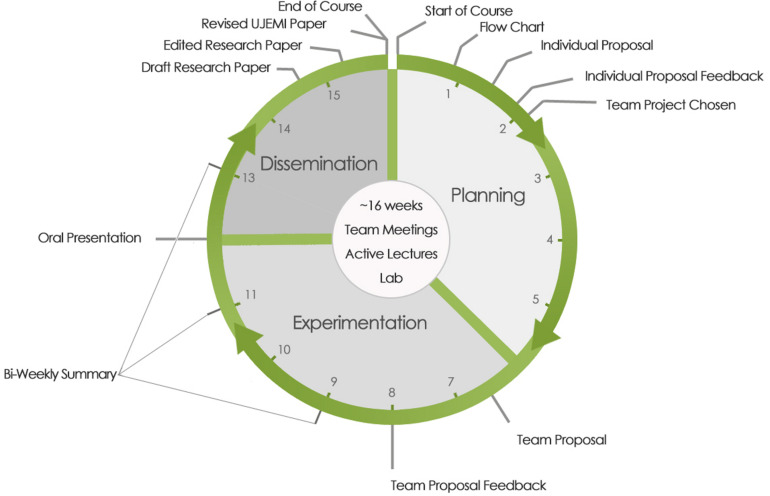
Research cycle over a 16 week academic term. Planning, experimentation, and dissemination phases are denoted. Due dates for communication assignments and feedback scaffolding the CURE are shown on the periphery. Individual proposals are submitted after the first week of classes. The team proposal is submitted at the conclusion of the planning phase in week 6. Teams conduct oral presentations on week 12 at the beginning of the dissemination phase. Draft manuscripts are submitted at the end of week 14 and the final manuscript just before the end of the course around week 15.

Our CURE is divided into three phases where students engage in planning, experimentation, and dissemination, respectively (Figure 1). Writing assignments are used to scaffold the process and provide clear milestones as the course unfolds.

The planning phase (weeks 1–6) begins by directing students to papers published in UJEMI by former students in the course. Students review the UJEMI literature and consider the data and proposed models. Students are encouraged to link their reading to the broader literature. Based on their reading, individual students submit a flowchart as well as a 1-page letter of intent explaining their proposed research question, hypothesis, experimental questions, and potential outcomes. Students also present a brief feasibility analysis. Written feedback on each proposal is provided by the instructor and teaching assistant(s) (week 2). Teaching assistants in the CURE are typically senior graduate students with backgrounds in microbiology and/or immunology who have a demonstrated aptitude for experimental research and teaching.

During the planning phase, students self-assemble into teams of 3 or 4 people and are assigned a weekly meeting time. Each team evaluates each individual proposal and selects a lead project to carry forward for the term. The team’s decision considers the potential scientific impact of the project, areas of development that individuals or the team would like to pursue (e.g., experience with a specific technique), feasibility, and the risk to reward ratio. The course learning objectives do not include a prescribed set of techniques that the students must learn; rather the focus is on working through a hypothesis-driven research project using the tools best suited to address the research question.

The team then moves into a series of team meetings in which the lead proposal is developed (weeks 2–6). The instructor and teaching assistants provide guidance as students refine their proposal to ensure that their hypothesis is testable, that their experiments are well designed and technically feasible, that they are sourcing reliable protocols and methods, and that they create strategies to execute their project within the constraints of the course (e.g., time, resources, expertise). It is important to note that the instructor and teaching assistants do not direct project development, but instead facilitate the process. The students are expected to guide their own research direction, which promotes project ownership. The development of a novel research question and self-directed approach to project management are elements of CUREs that have shown to increase student perceptions of ownership over their own projects and the outcomes associated with their projects (Cooper et al., 2019). This planning phase concludes with submission and feedback on an extensive team-based proposal that details the scientific background, hypothesis, experimental aims, protocols, and methods, laboratory safety considerations, a timeline, and pitfalls and workarounds (week 8). The team proposal becomes a road map for the project.

The experimentation phase is carried over weeks 6–12. Student teams are assigned a lab bay and prepare their own reagents including stock solutions and growth media. They also design primers and culture lab-based *Escherichia coli* strains which they can request from the course strain collection, the Coli Genetic Stock Center at Yale University, or from academic researchers around the world who have published strain descriptions. Students plan their own lab work schedules and are encouraged to divide the work amongst their team members so as not to over burden any one individual. The lab is open during the week from approximately 8a.m. to 5p.m. and students come and go during the day. Although we don’t monitor the time spent working on the project, student teams are given the same explicit deadlines. We estimate that individual students spend approximately 4–6 h per week working on their project which, if equitably distributed across their team, accounts for about 16–24 h of team-based lab work per week. Instructors and teaching assistants are available for guidance and demonstrations of technical steps. Similar to most research experiences, experiments rarely work on the first attempt and students often repeat steps before achieving a result. Bi-weekly written research reports and team meetings are used for reflection and feedback (weeks 9, 11, and 13). Students are often able to troubleshoot their own experiments after systematic reflection in written form. The experimentation phase concludes with an oral presentation to the class summarizing their research question and findings (week 12). Peer- and instructor-based feedback is gathered to support the dissemination phase.

The final phase involves dissemination of research results in the form of an original research article (weeks 12–16). Building off instructor and peer feedback from their oral presentation, as well as classroom activities in which strategies for drafting an original research manuscript are discussed, the students assemble their data as figures and tables and attempt to construct a coherent story. Instructors and teaching assistants provide guidance especially with deeper data analysis and reaching well-supported conclusions to provide students with enough scaffolding to facilitate the drafting process. Student teams submit a draft manuscript, formatted as per the Instructions for Authors guidelines set out by the Journal of Bacteriology. The manuscripts are reviewed by the instructor and teaching assistants (week 15) and returned to the student teams for revision (week 16). Students revise their work (often extensively) prior to final submission and provide a response to reviewers (week 16). A course grade is not assigned until the paper is accepted for publication in UJEMI. Students have the option of advancing their manuscript to a peer review phase if their work communicates a *bona fide* well-controlled finding (either negative or positive data). The peer review process extends beyond the end of the course (Sun et al., 2020). Importantly, papers published in UJEMI serve as fuel for the next iteration of the course, and the research cycle continues.

## Connecting Research Projects

**FIGURE 2:**
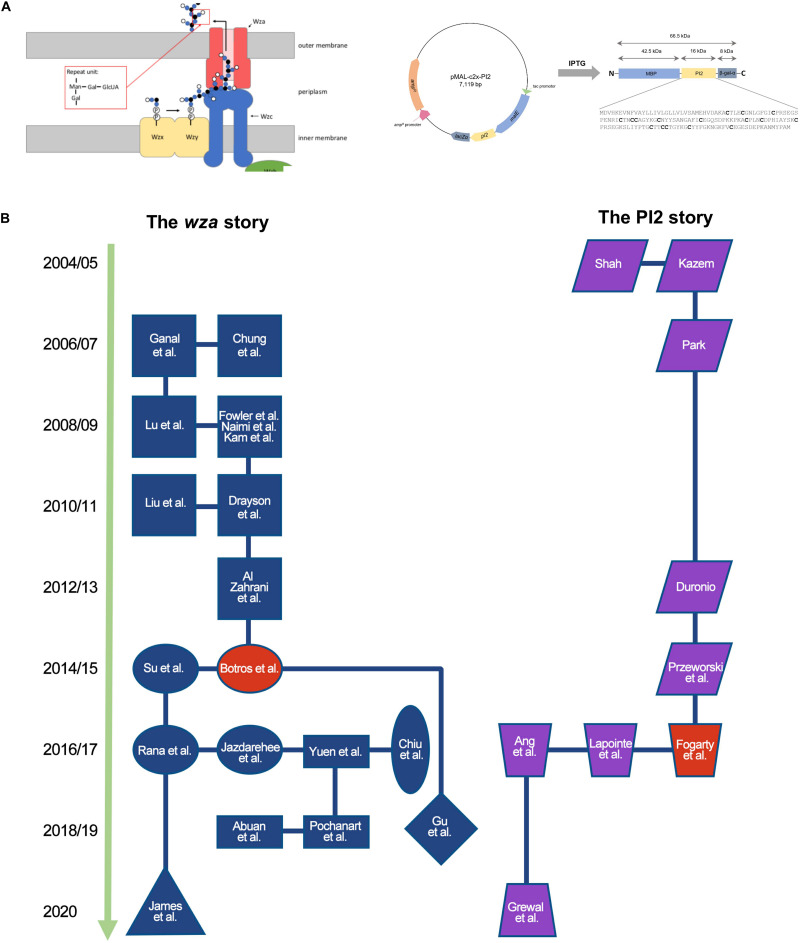
UJEMI case studies demonstrate how each CURE build on each other over time. **(A)** Left: working model of proteins involved in the secretion of capsule in *E. coli* strain K30 (reprinted with permission, Yuen et al., 2017). Gene products discussed include Wza (red), Wzb (green), Wzc (blue). Capsule subunits are shown in small blue and black circles. Auxiliary secretion machinery subunits (Wzx, Wzy) are not discussed in the text. Right: Plasmid map, PI2-MBP fusion protein domain architecture, and primary amino acid sequence of PI2 (reprinted with permission, Grewal et al., 2020). Cysteine residues are shown in bold font. **(B)** Chronology of ongoing CURE-based research projects published in UJEMI investigating the capsule on antibiotic resistance (left, blue/red project nodes) and the expression and purification of protease inhibitor 2 (PI2) (right, purple/red project nodes). Projects are labeled with the name of the first author in the corresponding UJEMI publication (refer to Supplementary Table S1). Projects sharing similar research aims are depicted as the same shape project node. The red project nodes represent research articles describing key advancements in each project.

Over 4 months, student teams work through a research cycle (Figure 1). The publication of a UJEMI article creates a body of knowledge that can be used to derive new research questions. A broad range of projects have been developed by students in the course that span the fields of molecular biology, biochemistry, and microbiology. Projects include research on bacteriophage (Chiu et al., 2017; Dimou et al., 2019), bacteria (Cramb et al., 2015; Backstrom et al., 2017; Hartstein et al., 2017), and yeast (Goldhawke et al., 2016), as well as *Caenorhabditis elegans* as a model host organism (James et al., 2018; Cheng et al., 2019). Students have employed a wide range of microbiology and molecular biology techniques including standard PCR, quantitative PCR, Gibson’s cloning, flow cytometry, and Next Generation Sequencing. As of 2019, UJEMI had published 493 original research articles solely authored by undergraduate students. Individual articles investigating common research questions can be clustered into ongoing course-based projects. Two ongoing research projects are summarized in Supplementary Table S1 and are mapped chronologically in Figure 2. We review these two projects as case studies to provide insight into how research evolves over multiple terms of the course.

## The *Wza* Story: Antibiotic Susceptibility and Capsule Secretion Genes

It has been suggested that capsule, a discrete layer of polysaccharide linked to the cell surface of some bacteria including *E. coli*, could create a physical barrier to impede the movement of molecules such as antibiotics into the cell (Slack and Nichols, 1982). Decreased intracellular concentration of the antibiotic may result in tolerance to high extracellular concentration of the antibiotic (i.e., increased resistance). Several mechanisms of capsule mediated resistance have been proposed including the idea that charged-based interactions between capsular polysaccharides and antibiotics may slow diffusion across the membrane (Slack and Nichols, 1982). Further, the regulation of capsule synthesis has been linked to stress response regulons in *E. coli* (Gottesman and Stout, 1991), leading to the notion that stress such as exposure to antibiotics may play a role in the regulation of capsule expression.

This project first began in our course when a student team decided to investigate the effects of sub-lethal doses of the antibiotics streptomycin and kanamycin on the synthesis of macromolecules in *E. coli* strain B23 (Chung et al., 2006). The students measured an increase in the concentration of hexose, a component of capsule, after treatment with the sub-lethal doses of the antibiotics (Chung et al., 2006). This study was followed up by two student teams who hypothesized that *E. coli* strain B23 treated with sub-lethal doses of kanamycin and streptomycin would increase capsule production (Ganal et al., 2007; Lu et al., 2008). The students found that capsule production increased following sublethal treatment with streptomycin and kanamycin (Ganal et al., 2007; Lu et al., 2008). However, follow-up studies were unable to link this phenotype with increased resistance to streptomycin (Fowler et al., 2009; Naimi et al., 2009), and increased resistance to kanamycin was observed in two studies (Kam et al., 2009; Al Zahrani et al., 2013), but not in a third (Drayson et al., 2011).

In 2014, the student team of Parmar et al. followed up on a report published in the journal Environmental Science and Pollution Research by researchers outside of the course suggesting that tetracycline interacts with capsular polysaccharides (Song et al., 2013). Parmar et al. asked whether capsule deficient mutants showed decreased resistance to tetracycline. The results of this study did not show a change in resistance to tetracycline (or streptomycin) in the capsular mutants (Parmar et al., 2014).

In 2014, the student team of Botros et al. initiated a new arm of the project by devising a screen to ask whether or not capsule contributes to resistance against a panel of 10 antibiotics representing different structural classes. The students drew upon the extensive research of Dr. Chris Whitfield at Guelph University in Canada whose lab has constructed defined deletion strains of the capsule secretion machinery in *E. coli* strain K30 (Figure 2A left; Whitfield, 2006). The students contacted Dr. Whitfield who generously provided wild type *E. coli* strain K30 and an isogenic strain (Δ*wza−wzb−wzc*) bearing a deletion of the genes encoding the outer membrane channel protein (Wza), the intermembrane ATPase (Wzb), and the inner membrane bound phosphatase (Wzc). Botros et al. developed a disk diffusion assay to semi-quantitatively compare the resistance of capsule deficient mutant strains and the wild type strain. After optimization, the disk diffusion assay was shown to be efficient and reliable. The results showed statistically significant differences in the zones of inhibition between the wild type and a capsule deficient mutant when treated with erythromycin and nitrofurantoin. Interestingly, resistance to erythromycin increased in the capsule deficient strain whereas resistance to nitrofurantoin decreased. Botros et al. chose to focus on the erythromycin result and followed up by showing that the phenotype was also observed for other macrolide antibiotics (e.g., clarithromycin, roxithromycin) but not for a ketolide (e.g., telithromycin). The student team concluded that deletion of the *E. coli* K30 group I capsule biosynthesis genes *wza, wzb*, and *wzc* confers capsule-independent resistance to macrolide antibiotics (Botros et al., 2015).

The next series of course projects utilized single gene deletion strains of Δ*wza*,Δ*wzb*,Δ*wzc* contributed again by Dr. Whitfield. After corroborating the results of Botros et al., student teams went on to show that deletion of *wza* is sufficient in conferring resistance to the macrolide erythromycin (Su et al., 2017) whereas deletion of *wzb* is not (Rana et al., 2016). Students also tested a Δ*wzc* deletion mutant which also showed a partially macrolide resistant phenotype (Jazdarehee et al., 2017).

The next set of student team projects asked whether complementation of *wza* in a strain bearing a deletion of this gene would restore the wild type (less erythromycin resistant) phenotype. The first attempt involved PCR amplification of the *wza* gene product and ligation into the TA TOPO cloning vector (Yuen et al., 2017). The student team of Yuen et al. designed PCR primers to amplify a product encompassing the putative *wza* promoter region to allow constitutive *wza* expression. The team obtained clones which they analyzed using Sanger sequencing. All of the inserts were found to be oriented in the same direction opposing the plasmid borne *lac* promoter sequence used for blue/white screening. The team surmised that the *wza* gene product may be lethal when overexpressed. Based on these results, the student team of Pochanart et al. decided to subclone the *wza* gene into a pBAD24 vector which encodes a promoter that can be upregulated and downregulated with the addition of media-based L-arabinose or glucose, respectively (Pochanart et al., 2018). The students were able to obtain clones which were verified by Sanger sequencing. Growth experiments showed that the high inducer concentration reduced the growth rate of the clones transformed with the *wza-*containing plasmid (Pochanart et al., 2018). This was consistent with the previous suggestion that overexpression of *wza* may be lethal (Yuen et al., 2017). The next student project set out to optimize the concentration of arabinose inducer to minimize the effect on growth rate (Abuan et al., 2018). After optimizing the inducer concentration, the students were able to show that in a strain bearing a deletion of *wza*, arabinose induction of a plasmid-encoded copy of *wza* was sufficient to restore erythromycin sensitivity of a Δ*wza* deletion strain using a disk diffusion assay (Abuan et al., 2018).

Students have started to explore the structure and function of the outer membrane channel protein Wza to understand how it is linked to the macrolide sensitivity. Using the crystal structure Wza (Dong et al., 2006), Su et al were able to measure the diameter, electrostatic properties and hydrophobicity of the pore. The students estimated the Wza pore to have a diameter of approximately 17 angstroms whereas the approximate size of erythromycin is 12 angstroms, suggesting that the channel may be sufficiently large enough to accommodate the antibiotic. The team acknowledged that electrostatic interactions and hydrophobicity of the Wza channel may also influence antibiotic movement through the channel. The student team of Chiu et al. (2017) followed up with a study that tested mutant specific tolerance to macrolides with different structural properties including erythromycin, clarithromycin, and roxithromycin, and telithromycin. The authors reported that *wza* linked resistance was observed for erythromycin, clarithromycin, and roxithromycin but not for telithromycin, the latter having distinctive aromatic rings and ketone groups. Chiu et al. (2017) speculated that the additional ketone groups on telithromycin may increase its polarity which may influence how it crosses the membrane relative to the other tested macrolides. Surprisingly, a single *wza* deletion mutant was shown to be more resistant than a Δ*wza−wzb−wzc* triple deletion mutant when treated with azithromycin, perhaps insinuating a more complex, structure-specific model of antibiotic uptake (Chui et al., 2017).

The students have proposed a range of models to explain how deletion of Δ*wza* renders *E. coli* strain K30 resistant to macrolides. Su et al. (2017) suggested a model in which Wza stabilizes other outer membrane proteins involved in outer membrane integrity. Botros et al. suggested that the formation of K-LPS in the absence of the capsule secretion genes alter the stability of permeability of the outer membrane (personal communication with Dr. Chris Whitfield, 46). Finally, several studies on the macrolide resistant phenotype linked to *wza* have observed that the effect is limited to experiments done on solid media (disk diffusion assays) as opposed to liquid media (broth dilution assays) (Rana et al., 2016; Jazdarehee et al., 2017; Su et al., 2017). How the nature of the growth media influences the observed phenotype remains an open question. The student team of James et al. (2020) have asked whether the discrepancy between experiments performed in liquid vs. solid phase media reflect a phenotype related to biofilm formation (James et al., 2020). While a compelling hypothesis, James et al. reported that their data showed no correlation between biofilm production in liquid media and erythromycin resistance in *E. coli* K30 wild-type, Δ*wza*, and Δ*wza−wzb−wzc* (James et al., 2020).

A recent study by the student team of Gu et al. (2018) revisited the initial data describing the antibiotic screen published by Botros et al. (2015). Gu et al. (2018) were specifically interested in the observation that a triple deletion of Δ*wza−wzb−wzc* results in a decreased resistance to the antibiotic nitrofurantoin. Following an extensive effort to verify the DNA sequence of each of the mutations in each strain, the students showed that deletion of *wzb* is sufficient to decrease resistance to nitrofurantoin. To explain their data, Gu et al. (2018) present a working model in which nitrofurantoin toxicity is reduced in the absence of the *wzb* phosphatase, possibly by increasing the concentration of a phosphorylated form of a putative reductase.

## The PI2 Story: Protein Expression and Disulphide Bond Formation

The production of recombinant protein in a functionally folded conformation is a long-standing challenge faced by many microbiologists and biotechnologists (Rosano et al., 2019). The expression of proteins containing disulphide bonds in prokaryotic organisms such as *E. coli* is confounded by the naturally occurring net reducing redox state of the cytosol (Ren et al., 2016). Interested in better understanding the function of the reductase protein domain thioredoxin (Trx) that has been shown to promote the solubility of fusion proteins containing disulphide bonds, Shah (2004) initiated a study within our CURE to investigate the effect of a Trx fusion on solubility of proteinase inhibitor 2 (PI2) from potatoes. PI2 is a relatively small 21 kDa, dimeric, cysteine-rich, heat-stable, endo-acting peptidase that inhibits chymotrypsin and trypsin protein containing 16 cysteine residues predicted to form 8 disulphide bonds (Keil et al., 1986). Using a plasmid containing the PI2 gene sequence that was donated to the course, several iterative attempts were made at cloning the gene into the pET32 expression vector (Invitrogen) (Kazem, 2004; Shah, 2004; Park, 2006; Duronio, 2012; Przeworski et al., 2015). The student team of Geum et al. eventually constructed a PI2-Trx fusion plasmid that was confirmed by restriction enzyme analysis, however, overexpression of the PI2-Trx protein product was not observed in whole cell lysates of *E. coli* strain BL21(DE3) using SDS-PAGE analysis stained with Coomassie blue. Geum et al. tentatively concluded that pET32 and/or strain BL21(DE3) may not be a suitable expression vector/host for overexpression of PI2. In section “Future directions,” the authors suggested Sanger sequencing to rule out mutations within their construct as well as Western blots as a more sensitive method of analysis (Geum et al., 2015).

In 2016, the student team of Fogarty et al. (2016) revisited the PI2 expression project. They began by using Sanger sequencing to determine the DNA sequence of the *pi2* insert and its genetic fusion to the thioredoxin domain (Fogarty et al., 2016). The authors analyzed the resulting DNA sequence to discover that the insert contained eukaryotic introns that resulted in a truncated protein due to an in-frame stop codon. The potato-derived *pi2* gene sequence also contained codons rarely used in *E. coli*. Fogarty et al. (2016) therefore adapted their project goal to design a version of the *pi2* sequence that lacked introns and was codon optimized for expression in *E. coli*. The team had their newly engineered DNA sequence synthesized as a gene block which they subcloned into a TOPO TA cloning plasmid. The next term, the student team of Lapointe et al. explored whether or not the newly designed *pi2* would be expressed when fused to either a maltose binding protein (MBP) domain or a hexahistidine tag (6XHis). The team subcloned the engineered *pi2* sequence from the TOPO TA plasmid construct into the commercially available pMALc2x and pET30b expression vectors that encode MBP and 6xHis tags, respectively (Figure 2A right). Expression analysis in BL21(DE3) transformed with each plasmid revealed a band in SDS-PAGE gels corresponding to the predicted molecular mass of PI2 fused to the MBP tag, although some protein degradation products were observed (Lapointe et al., 2016). Lapointe et al were the first to demonstrate PI2 expression and purification in our lab.

Ang et al. (2017) then opened a new branch of the project in our CURE by exploring whether or not altering the expression conditions or the cytosolic redox state of the *E. coli* expression host would impact PI2 expression levels. The authors compared PI2-MBP expression levels in *E. coli* strain Origami 2 (DE3) and *E. coli* wild type strain BL21(DE3). Origami 2 (DE3) bears mutations in glutaredoxin (*gor*) and thioredoxin (*trxB*) resulting in a net oxidizing cytoplasm. *E. coli* strain BL21 (DE3) encodes wild type copies of *gor* and *trxB* resulting in a net reducing cytoplasm. Contrary to their hypothesis predicting higher expression levels of the cysteine rich PI2 in *E. coli* strain Origami 2, SDS PAGE analysis of whole cell lysates showed over-expressed protein corresponding with the molecular mass of PI2-MBP in BL21(DE3) but not in Origami 2 (DE3) (Ang et al., 2017).

In 2019, the student team of Grewal et al. followed up by attempting to express PI2-MBP in *E. coli* strain SHuffle (C3028), which has a net oxidative cytoplasm (Lobstein et al., 2012; Grewal et al., 2020). Unlike Origami 2, SHuffle expresses a disulfide bond isomerase, DsbC, that facilitates proper protein folding by disrupting the formation of non-native disulfide bonds (Grewal et al., 2020). SDS PAGE analysis revealed a band that corresponds to the expected molecular mass of PI2-MBP. Using maltose affinity chromatography, the students purified a soluble form of PI2-MBP. They probed the tertiary structure of the protein using limited proteolysis and observed distinct bands indicative of a uniformly folded protein structure as opposed to an irregular aggregated protein. The team recommended follow up studies to further assess folding and function of purified PI2-MBP.

## Discussion

These two case studies describe a series of authentic scientific research projects that build on each other over time. Carried out by undergraduate student teams pursuing hypothesis-driven questions as part of a CURE, each individual research project focuses on novel investigations and original ideas that contribute to working biological models (Figure 2A). The two case studies follow distinct branching patterns which are defined by the results of experimentation and curiosity driven research questions depicted as nodes in Figure 2B.

Consistent with the use of original research articles as the conventional approach to the dissemination of research results in science, UJEMI articles serve as concise records of a series of small student-driven research projects that provide literature-based linkages between projects within the course. This model has been an effective approach to CURE development for several reasons. First, similar to a maturing grant-funded research laboratory, the accumulation of reagents, and scientific knowledge increases the power and efficiency of the ongoing research projects, which is motivating to students as it has the potential to yield more frequent, impactful, and exciting discoveries. Second, by focusing on novel research questions the participants engage in dynamic projects with broad meaning and relevance. In fact, UJEMI articles have been cited in articles published by well-established professional research journals (Chang et al., 2010 cited in Burmeister et al., 2020). Third, the UJEMI literature-base creates a “community of practice.” At the outset of the course, students are introduced to the journal as a repository of scientific investigations conducted by students who have come before them. Similar to any research project, they begin by “standing on the shoulders of giants” and they are expected to meet or exceed the effort and scientific rigor of their predecessors.

Each phase of the course uses UJEMI articles to facilitate student learning. In the planning phase, students read UJEMI papers, and derive new, follow-up research questions. In the experimentation phase, students experimentally verify the reliability of data in previous UJEMI papers looking for similarities and differences in results and interpretation before conducting novel analysis. In the dissemination phase, UJEMI articles are used as models for constructing a draft paper, as well as providing points for discussion. While the dissemination phase is notably short (i.e., 2–3 weeks to draft a manuscript), the students become familiar with the structure and function of UJEMI articles over the term before authoring their own manuscript. We surmise that by extensively working with the UJEMI articles in different contexts, the task of drafting a manuscript is made more efficient by indirectly scaffolding the writing phase with activities throughout the term that are linked to journal articles.

UJEMI articles provide students with concrete research topics and summaries of future directions, which enables student-driven project development by allowing the course instructor to provide arms-length verbal and written feedback to facilitate project development. In the planning phase, the instructor and teaching assistants provide written feedback on the individual proposal as well as the team proposal. The instructor and teaching assistants are also able to use team meetings to highlight aspects of previous studies that may impact the proposed research. Instructors often point out key papers in the field that the team should be aware of, known study limitations, and available research methods. The influence of the instructor on project development is more apparent in the dissemination phase when feedback is provided on the draft manuscript. Most often the instructor and teaching assistants work with the student authors to refine their paper in order to communicate evidence-based conclusions, clarify definitions, and explain ideas for future experiments that are both feasible and relevant. In cases where the research is communicated effectively in a UJEMI article, student teams tend to follow up with new research projects. If the research is communicated poorly, the projects tend to stall.

The prospect of being an author on a scientific manuscript is an aspect of our CURE model that promotes project ownership. Student authorship has previously been shown to benefit learning and research skill development in the context of CUREs (Cooper and Brownell, 2018; Corwin et al., 2018). All students participating in the course have the option of being included as an author on their team’s manuscript. The default approach to authorship order is alphabetically by last name; however, in some instances, teams have decided to change the authorship order to acknowledge specific contributions. To change from the alphabetically ordered authorship, all team members must approve. The team-based nature of the course, and co-authorship on a UJEMI publication, also promotes a sense of collaborative ownership of the project. For example, we observe a trend in our student’s written reflections about their research progress where they make statements moving from “…my project” to “…our project.” We hypothesize that systematic analyses of student reflections written over the course of the term will be a valuable metric to measure positive shifts in student confidence, ownership and the value of collaboration in the scientific process.

Analysis of individual UJEMI papers provides evidence of practices consistent with the notion of scientific enculturation. Case studies 1 and 2 include UJEMI articles that describe stages of scientific development in line with the outcomes of CURE participation predicted by Auchincloss et al. (2014). For example, writing the Introduction and Discussion sections requires content knowledge supported by credible scientific literature. The Methods and Materials section, as well as the Results section, capture a range of technical skill development, as well as collaboration skills as students share reagents within the course and interact with practicing scientists in the field. Since experimentation rarely follows a direct path, students learn to adapt their project goals and navigate uncertainty in their data. The conclusions sections of UJEMI papers reflect scientific maturity, as conclusions and claims are adjusted to more accurately reflect the data. Taken together, the process of doing authentic research through a journal-driven CURE means that students are fully immersed in the scientific experience. In order to meet the goal of publishing a scientific journal article, students need to engage with each of the CURE domains which comprehensively integrate the complex and dynamic processes underpinning the development of a scientist. A scientific project culminating in an original research article is an effective product to teach the process of doing science.

UJEMI articles are rich sources of objective data for understanding how our students are developing as scientists. A meta-analysis conducted by Linn et al. (2015) reported that more than half of 60 studies on undergraduate research experiences relied on subjective student-based, self-reporting surveys or interviews. The results of the study called for powerful and generalizable assessments to document student development to complement student surveys of perceived learning (Linn et al., 2015). Indeed, numerous studies have since described the development of validated and reliable survey instruments to assess student development in undergraduate research settings (Corwin et al., 2015; Shortlidge and Brownell, 2016; Ballen et al., 2017). Toward this end, we have collected preliminary data using the laboratory course assessment survey (LCAS) (Corwin et al., 2015) which showed student perceptions of learning aligned with the core domains of a CURE as outlined by Auchincloss et al. (2014). We are beginning to data mine and develop coding schemes for UJEMI articles to provide evidence of student learning within each domain of our CURE. One example is an assessment of scientific methodologies and skills developed as part of the CURE. Analysis of the Methods and Materials section of UJEMI articles provide evidence of techniques used by students in the course. Our preliminary data show that almost all student teams engage in *E. coli* strain isolation, PCR, Sanger Sequencing, and assay development/optimization. Since the projects are student-driven, the portfolio of techniques is not always predictable. Nevertheless, knowing what techniques are most commonly used helps the teaching team tailor scaffolding activities to guide student learning in the course. As a second example, evidence of collaboration can be gleaned from analyzing the Methods and Materials section as well as the Acknowledgments section of papers. Teams often recognize the contributions of other students in the course as well as researchers within and beyond the boundaries of our institution. Collaboration data informs course instructors of instances where scientific interactions can be fostered and better supported in future iterations of the course. We are also conducting deeper analyses of the writing assignments used to scaffold our CURE. Artifacts of learning, such as bi-weekly research summaries or research proposals, provide detailed accounts of activities including troubleshooting, reflection, and planning. We anticipate that additional meta-analyses of UJEMI papers, and associated writing assignments, will provide valuable integrated metrics of student development as scientists. Analyses over time will also provide dynamic perspectives reflecting the inner workings of our CURE to inform future curricula development to continually refine how best to meet the needs of our students.

The CURE described herein challenges students to develop and execute a novel research project with the goal of delivering a publication quality scientific manuscript in only 4 months. From the outset of the CURE, the students are made aware that their goal can be achieved by working as a team in a disciplined manner through a series of structured assignments that contribute to each research phase. A 2016 survey of alumni (*n* = 67) from our program showed that 93.9% of the respondents perceived their CURE experience as worthwhile, with 49 students (74.2%) indicating that the experience was “definitely” worthwhile and 13 students (19.7%) indicating that the experience was “somewhat” worthwhile. Three students (4.5%) indicated that the experience “had no value to them” and one student (1.5%) indicated that they felt the experience was “not a good use of their time.” These survey data were supported by comments which included these reflections from two alumni:

“*Being able to do a project from scratch with so much freedom is something that I have not yet seen in any other course, but I feel is extremely important and helpful. In addition to the learning, the freedom was quite thrilling, it provided a feel of what science really is like (lots of time reading papers and troubleshooting), rather than sitting in a lecture theatre memorizing what a professor says, or following step by step procedures for an experiment I may not completely understand or care about how well the results for it turned out.*”

“*Overall, I think it was a really valuable experience, as all the other lab courses are basically cookbook style courses and here we were able to figure things out for ourselves and research what we were interested in. […] it seemed a little daunting when starting the course, but it was really a lot of fun in the long run and I think I learned a lot.”*

These comments support the idea that student perceptions of learning align with the overall learning objectives of our CURE. Going forward we envision using mixed methods approaches combining validated survey instruments such as the LCAS, student reflections on learning, and coded analyses of learning artifacts such as UJEMI articles to better understand how CURE experiences can be designed and optimized to meet student needs.

## Conclusion

Our journal-driven CURE model provides students with an opportunity to engage in a disciplined experience that guides them through three critical phases of doing science: planning, experimentation, and dissemination. We depict these phases and their corresponding writing assignments as a cycle (Figure 1). Through iterative cycles of the course we have learned to appreciate the value of the time invested in each phase. A well-planned project with a testable hypothesis tends to provide concrete results and can be quite productive, especially in the context of a relatively short undergraduate course. The functional linkages between projects in the course underscore the value of the dissemination phase in terms of distilling information needed to carry on the project in another academic term. Further, the time constraint placed on the dissemination phase motivates students to summarize and communicate their findings in a timely manner. This model ostensibly reflects the process that most scientists would envision when taking on a new project; however, without structure, it is not uncommon for the planning and dissemination phases to be rushed or unbounded, respectively. Moreover, without formal milestones such as writing assignments, feedback critical for progressive development may be limited or absent altogether. We suggest that the research cycle model presented here may be useful, not only in CURE settings, but in other research settings in which trainees are developing including undergraduate internships or graduate studies.

## Data Availability Statement

The original contributions presented in the study are included in the article/Supplementary Material, further inquiries can be directed to the corresponding author.

## Ethics Statement

Written informed consent was obtained from the individual(s) for the publication of any potentially identifiable images or data included in this article.

## Author Contributions

DO and MG performed the conceptualization and acquisition of funding. DO prepared the original draft. All authors participated in the preparation and editing of this manuscript.

## Conflict of Interest

The authors declare that the research was conducted in the absence of any commercial or financial relationships that could be construed as a potential conflict of interest.

## Footnotes

1 https://ujemi.microbiology.ubc.ca/

## Supplementary Material

Supplementary Table 1 Metadata compilation of UJEMI articles discussed in the two case studies.
