# Supplementary material for: Propelling a Course-Based Undergraduate Research Experience Using an Open-Access Online Undergraduate Research Journal
Source: Front Microbiol. 2020 Nov 23;11:589025. doi: 10.3389/fmicb.2020.589025 (PMC7719674; doi:10.3389/fmicb.2020.589025)
Supplement: Supplementary Table 1 — Metadata compilation of UJEMI articles discussed in the two case studies. [file Table_1.pdf]

Supplementary Material

1 Supplementary Table

Table S1. Metadata compilation of UJEMI articles discussed in the two case studies.

**Case Study 1**			
**Year**	**Authors**	**Title of Paper**	**URL**
2006	Chung, Hung, Lam, Madera	Secondary effects of streptomycin and kanamycin on macromolecular composition of *Escherichia coli* B23 cell	https://www2.microbiology.ubc.ca/sites/default/files/roles/drupal_ungrad/JEMI/9/9-11.pdf
2007	Ganal, Gaudin, Roensch, Tran	Effects of streptomycin and kanamycin on the production of capsular polysaccharides in *Escherichia coli* B23 cells	https://www2.microbiology.ubc.ca/sites/default/files/roles/drupal_ungrad/JEMI/11/11-54.pdf
2008	Lu, Trinh, Tsang, Yeung	Effect of growth in sublethal levels of kanamycin and streptomycin on capsular polysaccharide production and antibiotic resistance in *Escherichia coli* B23	https://www.microbiology.ubc.ca/sites/default/files/roles/drupal_ungrad/JEMI/12/12-21.pdf
2009	Fowler, Hu, Hou, Wong	The effect of sub-inhibitory streptomycin on capsular polysaccharide production and streptomycin resistance in *Escherichia coli*	https://www.microbiology.ubc.ca/sites/default/files/roles/drupal_ungrad/JEMI/13/13-47.pdf
2009	Naimi, Nazer, Ong, Thong	The role of *wza* in extracellular capsular polysaccharide levels during exposure to sublethal doses of streptomycin	https://www.microbiology.ubc.ca/sites/default/files/roles/drupal_ungrad/JEMI/13/13-36.pdf
2009	Kam, Lou, Song	Effects of reduced capsular polysaccharide on kanamycin resistance in *Escherichia coli* B23 Cells	https://www.microbiology.ubc.ca/sites/default/files/roles/drupal_ungrad/JEMI/13/13-22.pdf
2010/11	Liu, Zhu, Zhu	persistence of antibiotic resistance and capsule in *E. coli* B23 after removal from sublethal kanamycin treatment	https://www.microbiology.ubc.ca/sites/default/files/roles/drupal_ungrad/JEMI/15/JEMI15_43-46.pdf
2011	Drayson, Leggat, Wood	Increased antibiotic resistance post-exposure to sub-inhibitory concentrations is independent of capsular polysaccharide production in *Escherichia coli*	https://www.microbiology.ubc.ca/sites/default/files/roles/drupal_ungrad/JEMI/15/JEMI15_36-42.pdf
2013	Al Zahrani, Huang, Lam, Vafaei	Capsule formation is necessary for kanamycin tolerance in *Escherichia coli* K-12	https://www.microbiology.ubc.ca/sites/default/files/roles/drupal_ungrad/JEMI/17/05.1G.pdf
2015	Botros, Mitchell, Van Ommen	Deletion of the *Escherichia coli* K30 group I capsule biosynthesis genes *wza, wzb* and *wzc*	https://jemi.microbiology.ubc.ca/sites/default/files/Botros%20et%20al..pdf

		confers capsule-independent resistance to macrolide antibiotics	
2016	Rana, Jang, Ahn, Nan	Single deletion of *Escherichia coli* K30 group I capsule biosynthesis system component, *wzb*, is not sufficient to confer capsule-independent resistance to erythromycin	https://jemi.microbiology.ubc.ca/sites/default/files/JEMI20-4.pdf
2017	Su, Wang, Yeo	Deletion of the group 1 capsular gene *wza* in *Escherichia coli* E69 confers resistance to the antibiotic erythromycin on solid media but not in liquid media	https://jemi.microbiology.ubc.ca/sites/default/files/Su%20et%20al%20JEMI%2B%20Vol%203%20pg%201-8.pdf
2017	Jazdarehee, Anderson, Morrison, Pardoe	Deletion of *Escherichia coli* K30 type I capsule assembly gene *wzc* confers resistance to the antibiotic erythromycin in solid media	https://jemi.microbiology.ubc.ca/sites/default/files/Jazdarehee%20et%20al%20JEMI%20Vol%2021%20pg%20108-112.pdf
2017	Yuen, Ting, Kang, Wong	Investigation of Wza in erythromycin sensitivity of *Escherichia coli* K30 E69 by genetic complementation	https://jemi.microbiology.ubc.ca/sites/default/files/Yuen%20et%20al%20JEMI%20Vol%2021%20pg%2052-57.pdf
2017	Chiu, Han, McCrystal, Zuo	Macrolide structures can confer differential susceptibility in *Escherichia coli* K30 deletions of group 1 capsule assembly genes	https://jemi.microbiology.ubc.ca/sites/default/files/Chiu%20et%20al%20JEMI%2B%20Vol%203%20pg%2050-56.pdf
2018	Pochanart, Richardson, Truong, Wang	Plasmid mediated complementation of *wza* in *Escherichia coli* K30 strain CWG281 restores erythromycin sensitivity	https://jemi.microbiology.ubc.ca/sites/default/files/Pochanart%20et%20al.%202018.pdf
2018	Abuan, AbuZuluf, Ban, Malekafzali	Plasmid-mediated complementation of *wza* restores erythromycin susceptibility in *Escherichia coli* K30 strain CWG281	https://jemi.microbiology.ubc.ca/sites/default/files/Abuan-JEMI%2BVol4%202018.pdf
2018	Gu, Khan, Pagulayan, Tam	Deletion of the capsule phosphatase gene *wzb* renders *Escherichia coli* strain K30 sensitive to the antibiotic nitrofurantoin	https://ujemi.microbiology.ubc.ca/sites/default/files/Gu-JEMI%2BVol4%202018.pdf
2020	James, Kim, Pan, Zhong	Biofilm production in *Escherichia coli* K30 with group 1 capsular gene *wza* and *wza-wzb-wzc* deletions is not correlated with erythromycin resistance phenotypes in liquid media	https://ojs.library.ubc.ca/index.php/UJEMI/article/view/193275/189971

Case Study 2

Year	Authors	Title of Paper	URL
2004	Shah	Preparing plasmid constructs to investigate the characteristics of thiol reductase and flavin reductase with regard to solubilizing insoluble proteinase inhibitor 2 in bacterial protein overexpression systems	https://www.microbiology.ubc.ca/sites/default/files/roles/drupal_ungrad/JEMI/6/6-20.pdf
2004	Kazem	Cloning EDTA monooxygenase as a model protein to characterize the effects of flavin	https://www.microbiology.ubc.ca/sites/default/files/roles/drupal_ungrad/JEMI/6/6-26.pdf

		oxidoreductase on solubility of proteins in protein overexpression systems	
2006	Park	Generation of recombinant plasmid constructs to assess the ability of NADH:flavin oxidoreductase to solubilize proteinase inhibitor 2 in bacterial protein overexpression systems	https://microbiology.ubc.ca/sites/default/files/roles/drupal_ungrad/JEMI/10/10-27.pdf
2012	Duronio	Production of a recombinant vector to enable the study of thioredoxin function as a bound or detached solubilizer of proteinase inhibitor 2 in a bacterial protein overexpression system	https://www.microbiology.ubc.ca/sites/default/files/roles/drupal_ungrad/JEMI/16/JEMI16_79-84.pdf
2015	Przeworski, Pham, Wang, Murillo	Attempted construction of recombinant vectors designed to study the solubility of overexpressed proteinase inhibitor 2 when co-expressed with thioredoxin	https://jemi.microbiology.ubc.ca/sites/default/files/Przeworski%20et%20al.pdf
2016	Lapointe, Li, Mortazavi, Zeng	Expression and purification of a potato type II proteinase inhibitor in *Escherichia coli* strain BL21(DE3)	https://jemi.microbiology.ubc.ca/sites/default/files/062916_Lapointe%20et%20al.pdf
2017	Ang, Atte, Halim, Jassal	Effect of temperature, inducer concentration, and *Escherichia coli* cytosolic redox state on MBP-PI2 expression	https://jemi.microbiology.ubc.ca/sites/default/files/Ang%20et%20al%20JEMI%20Vol%2021%20pg%2011-14.pdf
2017	Forgarty, Alimohammadi, Siu, Stachowiak	Synthesis, cloning, and sequencing of a codon optimized variant of proteinase inhibitor ii designed for expression in *Escherichia coli*	https://jemi.microbiology.ubc.ca/sites/default/files/Fogarty%20et%20al.pdf
2020	Grewal, Kim, Shi, Tong	Comparative expression of potato proteinase inhibitor type II in an oxidative versus reductive cytosolic environment of *Escherichia coli*	https://ojs.library.ubc.ca/index.php/UJEMI/article/view/193194/190013
